# ﻿Taxonomic revision of the Southeast Asian brook barb genus *Poropuntius* Smith, 1931 (Teleostei, Cyprinidae) with description of a new species from Vietnam

**DOI:** 10.3897/zookeys.1204.120873

**Published:** 2024-06-06

**Authors:** Huy Duc Hoang, Hung Manh Pham, Ngan Trong Tran, Jean-Dominique Durand, Ling Wu, John Pfeiffer, Xiao-Yong Chen, Lawrence M. Page

**Affiliations:** 1 Department of Ecology and Evolutionary Biology, University of Science, Ho Chi Minh City, Vietnam University of Science Ho Chi Minh City Vietnam; 2 Vietnam National University, Ho Chi Minh City, Vietnam Vietnam National University Ho Chi Minh City Vietnam; 3 IRD, MARBEC (University of Montpellier, CNRS, Ifremer, IRD), Montpellier, France University of Montpellier Montpellier France; 4 Southeast Asia Wildlife Biodiversity Research Group, Kunming Institute of Zoology, the Chinese Academy of Sciences, Kunming, Yunnan 650223, China Kunming Institute of Zoology, the Chinese Academy of Sciences Kunming China; 5 Southeast Asia Biodiversity Research Institute, Chinese Academy of Science, Yezin, Nay Pyi Taw 05282, Myanmar Southeast Asia Biodiversity Research Institute, Chinese Academy of Science Yezin Myanmar; 6 Department of Invertebrate Zoology, National Museum of Natural History, Smithsonian Institution, 10th and Constitution Avenue NW, Washington, DC 20560, USA National Museum of Natural History Washington, DC United States of America; 7 Florida Museum of Natural History, University of Florida, 1659 Museum Road, Gainesville, FL 32611, USA University of Florida Gainesville United States of America

**Keywords:** Cypriniformes, molecular systematics, phylogeny, *Poropuntiusanlaoensis* sp. nov.

## Abstract

Molecular data from samples encompassing 22 nominal species of *Poropuntius* indicate that the species-level diversity in the genus has been vastly overestimated, likely due to inadequate taxon and geographic sampling and reliance on morphological characters that vary intra-specifically. The latter includes discrete mouth morphologies related to alternate feeding strategies (ecomorphs) within populations. One new species is described, *Poropuntiusanlaoensis* Hoàng, Phạm & Trần, **sp. nov.**, and 17 synonyms of six valid species names of *Poropuntius*, *P.krempfi*, *P.alloiopleurus*, *P.huangchuchieni*, *P.laoensis*, *P.kontumensis*, and *P.deauratus*, are recognised. Additional taxonomic changes in this widespread and generally poorly known genus are likely as more molecular and morphological data become available.

## ﻿Introduction

Thirty-three names currently are recognised as valid for species of *Poropuntius* (Fricke et al. 2023) with distribution of the genus ranging from the Irrawaddy River basin in Myanmar to the Mekong and Red River basins in Yunnan, China and south through Vietnam, Laos, Cambodia, Thailand, and peninsular Malaysia to Sumatra, Indonesia. The greatest diversity occurs in the Mekong basin of Yunnan and Vietnam, and only one species is known from peninsular Malaysia and one from Sumatra (Fricke et al. 2023).

*Poropuntius* has been characterised as usually having 8½ branched dorsal rays, large serrae on the posterior edge of the last simple dorsal ray, and tubercles covering the tip of the snout and occurring on the lacrimal bones (Rainboth 1996; Roberts 1998). Tubercles occur on both sexes and, at least in most species, on juveniles as well as adults (Roberts 1998). In addition, most species have rostral and maxillary barbels, a black to dusky submarginal stripe on the upper and lower caudal-fin lobes, and a well-developed keratinised edge on the lower jaw.

Considerable confusion surrounds the taxonomy of *Poropuntius*, with most species having been diagnosed using morphological characters that tend to be highly plastic and comparisons made to few congenerics. Wu et al. (2013) and Muhammad-Rasul et al. (2018) investigated molecular diversity of *Poropuntius* and, although they targeted only a few species and small geographic areas, their results suggested some morphological hypotheses concerning species delimitations are incorrect, and larger molecular studies are needed to improve our understanding of species diversity in *Poropuntius*. Recent fieldwork throughout much of the distribution of *Poropuntius*, especially in species-rich Vietnam, has provided material for a broader study of molecular variation in the genus. Results of that study, reported herein, suggest that several species names, including nominal species of *Acrossocheilus* and *Hypsibarbus* in Vietnam, are synonyms of previously described species. A newly discovered species from Vietnam is described using morphological and molecular characteristics.

## ﻿Materials and methods

### ﻿Field sampling

**Figure 1.**
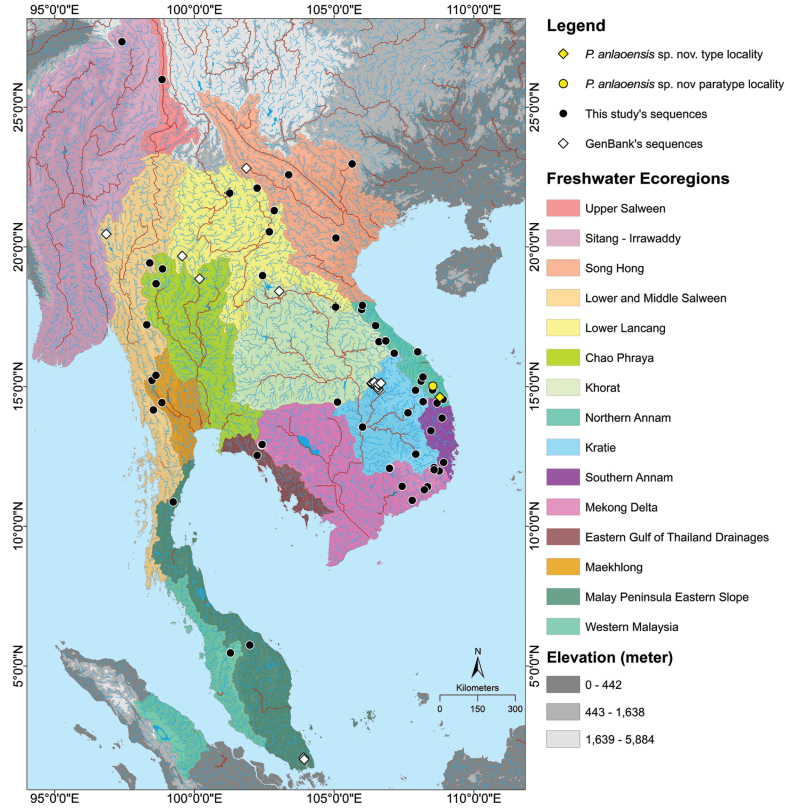
Sampling sites of *Poropuntius* in this study in 15 freshwater ecoregions shown in various colours.

Tissues were taken from selected specimens collected in the field or purchased in local markets throughout much of the range of *Poropuntius* (Fig. 1) and stored in 95% ethanol for molecular analysis. Specimens were then fixed in 10% formalin, subsequently transferred to 70% ethanol, and deposited at the University of Science, Ho Chi Minh City, Vietnam (**UNS**), Florida Museum of Natural History, Florida, USA (**UF**), and Kunming Institute of Zoology, Yunnan, China (**KIZ**). Samples newly obtained for this study included those from the same drainages as type localities for all nominal species in the molecular analysis except *P.krempfi* (Pellegrin & Chevey, 1934) for which samples were from near the type locality in the Red River drainage (Suppl. material 1: table S1). Multiple samples and localities were included, when possible, for widespread species. Sampling sites were assigned to freshwater ecoregions following Abell et al. (2008) and Hoang et al. (2021a, 2021b).

### ﻿Molecular analysis

Molecular sequence data were newly generated or taken from GenBank for the following currently recognised species of *Poropuntius* (Fricke et al. 2023): *P.alloiopleurus* (Vaillant, 1893), *P.aluoiensis* (Nguyen, 1997), *P.angustus* Kottelat, 2000, *P.bantamensis* (Rendahl, 1920), *P.bolovenensis* Roberts, 1998, *P.burtoni* (Mukerji, 1933), *P.carinatus* (Wu & Lin, 1977), *P.deauratus* (Valenciennes, 1842), *P.genyognathus* Roberts, 1998, *P.hampaloides* (Vinciguerra, 1890), *P.hathe* Roberts, 1998, *P.heterolepidotus* Roberts, 1998, *P.huangchuchieni* (Tchang, 1962), *P.kontumensis* (Chevey, 1934), *P.krempfi* (Pellegrin & Chevey, 1934), *P.laoensis* (Günther, 1868), *P.melanogrammus* Roberts, 1998, *P.normani* Smith, 1931, *P.opisthopterus* (Wu, 1977), *P.rhomboides* (Wu & Lin, 1977), *P.schanicus* (Boulenger, 1893), and *P.yalyensis* (Nguyen, 2001). Sequence data also were included for the following species considered to be related to *Poropuntius* and for which taxonomic ambiguity exists (Wu et al. 2013; Yang et al. 2015; Kang et al. 2016; Zheng et al. 2016; Muhammad-Rasul et al. 2018): *Poropuntiusbaolacensis* (Nguyen, 2001), *P.brevispinus* (Nguyễn & Đoàn, 1969), *Acrossocheilusxamensis* Kottelat, 2000, *A.macrophthalmus* (Nguyen, 2001), *Hypsibarbusannamensis* (Pellegrin & Chevey, 1936), and *H.macrosquamatus* (Mai, 1978). *Hypsibarbuswetmorei* was used at the outgroup species based on recent studies of cyprinid higher-level relationships (Yang et al. 2015; Stout et al. 2016; Wang et al. 2016). Given taxonomic ambiguities, it is important to identify methods used to identify specimens newly acquired by the authors for this study.

Specimens of *Acrossocheilusbaolacensis* Nguyen, in Nguyễn and Ngô 2001 (recently as *Poropuntiusbaolacensis*) was described from Vietnam, Cao Bang Province, Nho Que, Bao Lac, Song Hong ecoregion. Near-topotypic material (UNS 2018-1410) was collected in the Gâm River of the Song Hong ecoregion and identified through comparison with the description in Nguyễn and Ngô (2001: 385–387).

*Acrossocheilusmacrophthalmus* Nguyễn & Ngô, 2001 was described from Vietnam, Hòa Bình, Song Hong ecoregion, Black River. Near-topotypic material (UNS 2018-1310) was collected in the Gâm River of the Song Hong ecoregion and identified through comparison with the description in Nguyễn and Ngô (2001).

*Acrossocheilusxamensis* Kottelat, 2000 was described from Laos, Houaphan Province, Houay Tangoua, Nậm Xảm. Near-topotypic material (UNS 2019-1506) was collected from Vietnam, Thanh Hóa, Quan Hóa, Lò River, Song Hong ecoregion, in the same drainage as the type locality. The identification followed Kottelat (2000: 38–39).

*Acrossocheilusyalyensis* Nguyen, in Nguyễn and Ngô 2001 (recently as *Poropuntiusyalyensis*) was described from Vietnam, Kon Tum Province, Sesan River. Topotypic material (UNS 2019-1201) was collected from the Sesan River, Kon Tum, Dak Poko - Dak Pek, in the Kratie-Stung Treng ecoregion and identified through comparison with the description in Nguyễn and Ngô (2001: 393–395).

*Barbodeshuangchuchienirhomboides* Wu & Lin (in Wu et al. 1977: 248; recently as *Poropuntiusrhomboides*) was described from China, Yunnan Province, Yuanjiang (upper Red River). Near-topotypic material (UNS 2018-3010, UNS 2018-1111) was collected in the upper Red River in Vietnam, Lào Cai, Bản Hồ, Nậm Củn, and identified through comparison with the description in Wu et al. (1977).

*Barbusalloiopleurus* Vaillant, 1893 (now *Poropuntiusalloiopleurus*) was described from Vietnam, Đà River, Song Hong ecoregion. Topotypic material (UNS 2018-3010) was collected from the Đà River and identified through comparison with the description in Kottelat (2001: 35–36).

Barbus (Puntius) annamensis Pellegrin & Chevey, 1936 (recently as *Hypsibarbusannamensis*) was described from Vietnam, Annam, Quảng Nam, Hàn Giang. Topotypic material (UNS 2018-2702) and near-topotypic material (UNS 2018-2402, UNS 2018-0704) from the northern Annam ecoregion was identified following Rainboth (1996: 55–58).

*Barbusbantamensis* Rendahl, 1920 (recently as *Poropuntiusbantamensis*) was described from Thailand, Chiang Mai Province, Ban Tam. Topotypic material was collected in Thailand, Chiang Mai Province, Ping River drainage in the Chao Phraya ecoregion (UF 183366, UF 243217, UF 177816) and identified through comparison with the description in Rendahl (1920: 1–3).

*Lissochilusaluoiensis* Nguyen, 1997 (recently as *Poropuntiusaluoiensis*) was described from Vietnam, Thua Thien Hue Province, A Luoi, A Sap at Nham, Se Kong basin. Topotypic material (UNS 2018-0304) was collected from the A Sap River of the upper Sekong drainage and identified through comparison with the description in Nguyen (1997: 1–4).

*Lissochilusbrevispinus* Nguyễn & Đoàn, 1969 (recently as *Poropuntiusbrevispinus*) was described from Vietnam, Hoa Binh Province, Suối Rút. Material (UNS 2018-1310, UNS 2018-1010) was collected from Hà Giang, Lô River, in the Song Hong ecoregion and identified through comparison with the description Nguyễn and Ngô (2001: 387–388).

*Lissochilusmacrosquamatus* Mai, 1978 (recently as *Hypsibarbusmacrosquamatus*) was described from Vietnam, Song Hong ecoregion. Near-topotypic material (UNS 2018-1204, UNS 2018-1604) was collected in the Long Đại River, Quảng Bình of the northern Annam ecoregion and identified through comparison with the description in Mai (1978: 99–100).

*Poropuntiusangustus* Kottelat, 2000 was described from Laos, Louangphabang Province: Houay Houn, Ban Houay Lek. Topotypic material (UNS 2018-07-11) was collected from the Nam Nứa, Lower Lancang ecoregion, in the upper reach of Nam Ou, the type locality, and identified through comparison with the description in Kottelat (2000: 46).

*Poropuntiusbolovenensis* Roberts, 1998 was described from Laos, Champasak Province, Bolavens, Plateau, Sekong basin, Xe Nam Noi. Topotypic material was collected from Xe Nam Noi and Xe Pian in the Kratie-Stung Treng ecoregion and identified through comparisons with descriptions in Roberts (1998: 124–127) and Kang et al. (2016).

*Poropuntiuskrempfi* (Pellegrin & Chevey, 1934) was collected in the Gâm River of the Song Hong ecoregion. Identification of our specimens (UNS 2018-1410) was based on comparison with material from the Red River of Song Hong, northern Vietnam, the type locality.

*Poropuntiusnormani* Smith, 1931 was described from Thailand, Chantaburi Province, Pliew Waterfall. Topotypic material (UF 235973) and near-topotypic material (UF 170368, 188709, 235982) was collected from Chantaburi Province, in the Eastern Gulf of Thailand Drainages ecoregion and identified through comparisons with descriptions in Smith (1931: 15) and Muhammad-Rasul et al. (2018: 327–342).

No genetic data are available for the following species, and no opinions are offered on their validity: *Poropuntiuschondrorhynchus* (Fowler, 1934), *P.chonglingchungi* (Tchang, 1938), *P.cogginii* (Chaudhuri, 1911), *P.exiguus* (Wu & Lin, 1977), *P.faucis* (Smith, 1945), *P.fuxianhuensis* (Wang, Zhuang & Gao, 1982), *P.margarianus* (Anderson, 1879), *P.shanensis* (Hora & Mukerji, 1934), *P.speleops* (Roberts, 1991), and *P.tawarensis* (Weber & de Beaufort, 1916).

### ﻿DNA extraction and amplification

DNA was extracted from fin clips stored in 95–99% ethanol using the DNeasy Blood & Tissue Kit (Qiagen, Valencia, CA, USA) and following the protocol suggested by the manufacturer. Two mitochondrial genes, cytochrome oxidase subunit I (COI) and cytochrome b (Cyt*b*), were amplified using polymerase chain reaction (PCR). Primers and PCR conditions followed Ward et al. (2005) for COI and Durand et al. (2012) for Cyt*b.*PCR products were visualised on 1–2% agarose gels, and the most intense products were selected for purifying and Sanger sequencing by 1ST BASE (https://base-asia.com/).

### ﻿Phylogenetic analyses

Chromas 2.6.6 (http://technelysium.com.au/) was used to inspect the sequence chromatograms and assemble them into contigs, and MUSCLE in MEGA 7 (Edgar 2004; Kumar et al. 2016) was used to align the consensus sequences for each gene. Alignments were inspected by eye for accuracy, and sequences were trimmed at the 3’ and 5’ ends to minimise missing characters. The final data matrix consisted of 568 bp for COI and 872 bp for Cyt*b* used in the separated analyses. Uncorrected pairwise sequence divergence was estimated using the substitution model of Kimura 2-parameters, bootstraps 1000 implemented in MEGA 7 (Kumar et al. 2016). All sequences generated for this study were deposited in GenBank (Suppl. material 1: table S1). For each independent dataset of COI and Cyt*b*, phylogenetic inferences based on Maximum Likelihood (ML) were made using IQ-TREE (Nguyen et al. 2015) through the IQ-TREE web server (Trifinopoulos et al. 2016, http://www.iqtree.org/). Optimal partitioning models for the ML inference were selected by ModelFinder (Chernomor et al. 2016; Kalyaanamoorthy et al. 2017) in IQ-TREE, using the minimum BIC score. Partition analysis suggested best fit models for ML inference: TN+F+I+G4 (BIC = 6531.319, lnL = - 2650.474) for COI and HKY+F+I+G4 (BIC = 6910.503, lnL = - 3065.931) for Cyt*b.* Ultrafast bootstrap (BS) analysis for 1000 iterations (Bui et al. 2013) was carried out to determine statistical support for the nodes in ML. The trees obtained from ML were visualised using Figtree v. 1.4.3 (http://tree.bio.ed.ac.uk/software/figtree).

### ﻿List of abbreviations

**BD** body depth;

**BIC** Bayesian information criterion;

**BS** bootstrap;

**COI** Cytochrome *c* oxidase subunit 1;

**Cyt*b*** Cytochrome *b*;

**DCP** depth of caudal peduncle;

**DNA** Deoxyribonucleic Acid;

**HL** head length;

**LCP** length of caudal peduncle;

**ML** Maximum Likelihood;

**PCR** Polymerase Chain Reaction;

**SL** standard length.

## ﻿Results

**Figure 2.**
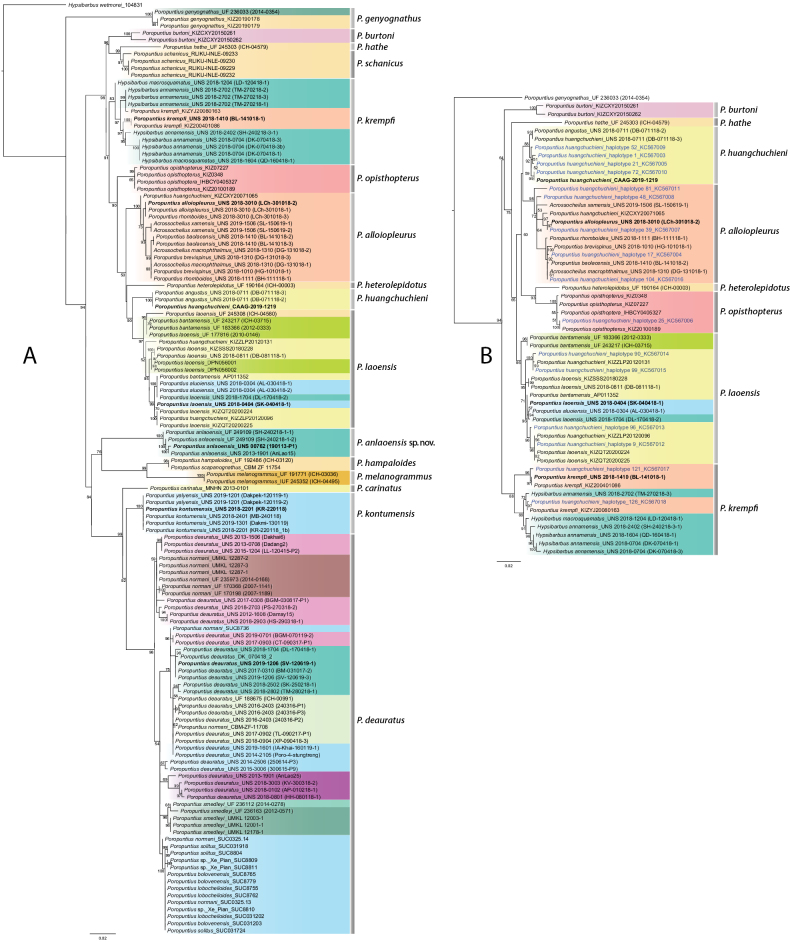
Maximum-likelihood tree based on (**A**) COI and (**B**) Cyt*b* mitochondrial gene sequences for species of *Poropuntius*. Numbers on branches are ML bootstrap values (values > 50% shown). Bold sample labels are sequences from type localities. Blue sample labels in the Cyt*b* tree are sequences from Wu et al. (2013). Colours of clades correspond to the freshwater ecoregions in Fig. 1.

The ML trees for the COI and Cyt*b* sequences are shown in Fig. 2. The COI topology is consistent with recognition of 16 species (Fig. 2A), all represented by more than one sequence with ≥ 83% bootstrap support, including one undescribed species.

The Cyt*b* sequences were used to examine the results of the study of *P.huangchuchieni* in southwest China by Wu et al. (2013) with a greater Cyt*b* dataset (Fig. 2B). The five major lineages identified by Wu et al. (2013) are reassigned. The LX lineage of Wu et al. (2013) from the Song Hong aligned with *P.krempfi* in our sequences, the SW of the Upper Salween with *P.opisthopterus*, the RL of the Song Hong with *P.alloiopleurus*, the MK-A of the Lower Lancang with *P.huangchuchieni*, and the MK-B of the Lower Lancang with *P.laoensis*. The greatest similarity in the Cyt*b* sequences was 98% (±0.00), found between *P.laoensis*, which occurs in the Lower Lancang, Kratie – Stung Treng, northern Annam, Chao Phraya, and Middle & Lower Salween ecoregions, and *P.huangchuchieni* in the Lower Lancang (Table 1). The COI data for *P.huangchuchieni* (Fig. 2A) aligned with the reassignments based on the Cyt*b* data and are not discussed further.

**Table 1.** Cytochrome *b* genetic distances between species in the phylogenetic analysis with *P.genyognathus* as the outgroup using the Kimura 2-parameter model, standard error estimates shown above the diagonal with bootstraps 1000.

	1	2	3	4	5	6	7	8	9
1. *P.genyognathus*		0.01	0.01	0.01	0.01	0.01	0.01	0.01	0.01
2. *P.burtoni*	0.09		0.01	0.01	0.01	0.01	0.01	0.01	0.01
3. *P.hathe*	0.10	0.06		0.01	0.01	0.01	0.01	0.01	0.01
4. *P.huangchuchieni*	0.08	0.05	0.05		0.00	0.01	0.01	0.00	0.01
5. *P.alloiopleurus*	0.08	0.06	0.05	0.03		0.01	0.01	0.01	0.01
6. *P.heterolepidotus*	0.09	0.06	0.06	0.03	0.04		0.01	0.01	0.01
7. *P.opisthopterus*	0.09	0.06	0.06	0.04	0.04	0.03		0.01	0.01
8. *P.laoensis*	0.08	0.05	0.05	0.02	0.03	0.04	0.04		0.01
9. *P.krempfi*	0.08	0.05	0.06	0.04	0.05	0.05	0.04	0.04	

Seven of the 16 species recognised with COI data (Fig. 2A) include representatives of other nominal species now to be recognised as synonyms. One, the *P.krempfi* clade includes 12 specimens of three nominal species currently assigned to two genera: *P.krempfi*, *Hypsibarbusannamensis*, and *H.macrosquamatus*. The newly collected specimens of all three nominal taxa were collected from their type localities in the Song Hong and northern Annam ecoregions and are embedded with *P.krempfi* specimens from the Song Hong with a mean similarity of 98.8% (± 0.29) (Fig. 2A). This clade received 83% bootstrap support. *Hypsibarbusannamensis*, and *H.macrosquamatus* are synonyms of *P.krempfi*. The *P.krempfi* lineage in the Song Hong ecoregion has a COI sequence similarity of 97% (± 1.00) with *P.alloiopleurus* from the Song Hong, *P.opisthopterus* from the Upper Salween, *P.burtoni* from the Sittang - Irrawaddy, and *P.schanicus* from the Inle Lake, Myanmar (Table 2).

**Table 2.** COI genetic distances between species in the phylogenetic analysis with *Hypsibarbuswetmorei* as the outgroup using the Kimura 2-parameter model, standard error estimates shown above the diagonal with bootstraps 1000.

	1	2	3	4	5	6	7	8	9	10	11	12	13	14	15	16	17
1. *H.wetmorei*		0.02	0.01	0.02	0.01	0.01	0.02	0.02	0.02	0.02	0.01	0.02	0.02	0.02	0.02	0.02	0.02
2. *P.genyognathus*	0.10		0.01	0.01	0.01	0.01	0.01	0.01	0.02	0.01	0.01	0.01	0.02	0.02	0.01	0.01	0.02
3. *P.burtoni*	0.08	0.08		0.01	0.01	0.01	0.01	0.01	0.01	0.01	0.01	0.01	0.01	0.01	0.01	0.01	0.01
4. *P.hathe*	0.10	0.09	0.04		0.01	0.01	0.01	0.01	0.01	0.01	0.01	0.01	0.01	0.01	0.01	0.01	0.01
5. *P.schanicus*	0.09	0.07	0.03	0.03		0.01	0.01	0.01	0.01	0.01	0.01	0.01	0.01	0.01	0.01	0.01	0.01
6. *P.krempfi*	0.09	0.08	0.03	0.05	0.03		0.01	0.01	0.01	0.01	0.01	0.01	0.01	0.01	0.01	0.01	0.01
7. *P.opisthopterus*	0.10	0.09	0.04	0.05	0.03	0.03		0.01	0.01	0.01	0.01	0.01	0.01	0.01	0.01	0.01	0.01
8. *P.alloiopleurus*	0.11	0.08	0.04	0.05	0.04	0.03	0.03		0.01	0.01	0.01	0.01	0.01	0.02	0.01	0.01	0.01
9. *P.heterolepidotus*	0.12	0.10	0.04	0.06	0.05	0.04	0.03	0.04		0.01	0.01	0.01	0.01	0.02	0.01	0.01	0.01
10. *P.huangchuchieni*	0.10	0.07	0.05	0.06	0.05	0.04	0.03	0.03	0.04		0.01	0.01	0.01	0.01	0.01	0.01	0.01
11. *P.laoensis*	0.09	0.07	0.04	0.05	0.04	0.04	0.03	0.03	0.04	0.03		0.01	0.01	0.01	0.01	0.01	0.01
12. *P.anlaoensis* sp. nov.	0.12	0.09	0.08	0.09	0.07	0.08	0.08	0.09	0.08	0.09	0.08		0.01	0.01	0.01	0.01	0.01
13. *P.hampaloides*	0.12	0.10	0.07	0.09	0.07	0.07	0.07	0.08	0.07	0.07	0.07	0.07		0.01	0.01	0.01	0.01
14. *P.melanogrammus*	0.12	0.10	0.09	0.09	0.08	0.08	0.09	0.10	0.10	0.08	0.08	0.09	0.05		0.02	0.02	0.01
15. *P.carinatus*	0.11	0.07	0.06	0.08	0.06	0.06	0.06	0.05	0.07	0.05	0.05	0.07	0.09	0.10		0.01	0.01
16. *P.kontumensis*	0.11	0.09	0.06	0.08	0.05	0.06	0.05	0.06	0.07	0.05	0.05	0.06	0.08	0.09	0.03		0.01
17. *P.deauratus*	0.11	0.10	0.07	0.08	0.06	0.07	0.06	0.07	0.08	0.07	0.07	0.07	0.08	0.09	0.04	0.04	

The *P.alloiopleurus* clade (Fig. 2A, B) consists of 13 specimens of six nominal species (plus specimens labelled *P.huangchuchieni* in Wu et al. (2013) and Zheng et al. (2016)), currently in two genera: *P.alloiopleurus*, *P.baolacensis*, *P.brevispinus*, *P.rhomboides*, *Acrossocheilusmacrophthalmus*, and *A.xamensis* collected in the Song Hong and northern Annam ecoregions from their type drainages (Fig. 2A). The newly collected specimens are embedded with *P.alloiopleurus* specimens from the Song Hong and show a mean similarity of 99.5% (± 0.20). This clade receives 100% bootstrap support. Thus, we conclude that *P.baolacensis*, *P.brevispinus*, *P.rhomboides*, *A.macrophthalmus* and *A.xamensis* are synonyms of *P.alloiopleurus*. The *P.alloiopleurus* lineage observed in the Song Hong ecoregion has a COI sequence similarity of 97% (±1.00) with *P.krempfi* from the Song Hong, *P.huangchuchieni* and *P.laoensis* from the Lower Lancang, and *P.opisthopterus* from the Upper Salween (Table 2).

The *P.huangchuchieni* clade consists of three specimens of two nominal species, *P.huangchuchieni* and *P.angustus* collected from their type drainages in the Lower Lancang ecoregion. These specimens have a mean similarity of 100% (± 0.00) (Fig. 2A). *Poropuntiusangustus* is a synonym of *P.huangchuchieni*. The *P.huangchuchieni* lineage in the Lower Lancang ecoregion has a COI sequence similarity of 97% (± 1.00) with *P.laoensis* from the Lower Lancang, *P.alloiopleurus* from the Song Hong, and *P.opisthopterus* from the Upper Salween (Table 2).

The *P.laoensis* clade consists of 17 specimens of three nominal species, *P.laoensis*, *P.aluoiensis*, and *P.bantamensis* collected in the Lower Lancang, Kratie – Stung Treng, northern Annam, Chao Phraya, and Middle & Lower Salween ecoregions including from their type localities. The newly collected specimens are embedded with *P.laoensis* specimens from the Kratie – Stung Treng and show a mean similarity of 99.1% (± 0.23) (Fig. 2A). This clade receives 97% bootstrap support. *Poropuntiusaluoiensis* and *P.bantamensis* are synonyms of *P.laoensis*. The *P.laoensis* lineage observed in the Lower Lancang, Kratie – Stung Treng, northern Annam, Chao Phraya, and Middle & Lower Salween ecoregions has a COI sequence similarity of 97% (± 1.00) with *P.huangchuchieni* from the Lower Lancang, *P.alloiopleurus* from the Song Hong, and *P.opisthopterus* from the Upper Salween (Table 2).

The *P.anlaoensis* sp. nov. lineage consists of four specimens of a new species of *Poropuntius*. This lineage from the Annam ecoregion has its greatest COI sequence similarity of 94% (± 1.00) with *P.kontumensis* from the Kratie – Stung Treng (Table 2).

The *P.kontumensis* clade consists of six specimens of two nominal species, *P.kontumensis* and *P.yalyensis*, collected in the Kratie – Stung Treng ecoregion from their type localities. The newly collected specimens are embedded with *P.kontumensis* specimens from the type locality and have a similarity of 100% (± 0.00) (Fig. 2A). *Poropuntiusyalyensis* is a synonym of *P.kontumensis*. The *P.kontumensis* lineage from the Kratie – Stung Treng ecoregion is most similar to *P.carinatus* from the Lower Lancang with a COI sequence similarity of 97% (± 1.00) (Table 2).

The *P.deauratus* clade consists of 57 specimens of six nominal species, *P.normani*, *P.deauratus*, *P.bolovenensis*, *P.lobocheiloides*, *P.smedleyi*, and *P.solitus*, collected the eastern Gulf of Thailand drainages, Malay Peninsula, Mekong Delta, Kratie – Stung Treng, Khorat Plateau, and Annam ecoregions, including from their type localities. These specimens are embedded with *P.deauratus* specimens from the Annam ecoregion and have a mean similarity of 98.7% (± 0.25). This clade receives 98% bootstrap support. *Poropuntiusnormani*, *P.bolovenensis*, *P.lobocheiloides*, *P.smedleyi*, and *P.solitus* are synonyms of *P.deauratus*. The *P.deauratus* lineage in the Eastern Gulf of Thailand drainages, Malay Peninsula, Mekong Delta, Kratie – Stung Treng, Khorat Plateau, and Annam ecoregions has a COI sequence similarity of 96% (± 1.00) with *P.carinatus* from the Lower Lancang and *P.kontumensis* from the Kratie – Stung Treng ecoregion (Table 2).

### ﻿Taxonomic conclusions

Based on molecular data, 17 nominal species of *Poropuntius*, *Hypsibarbus*, and *Acrossocheilus* are reduced to synonyms of the six valid species of *Poropuntius* listed below. Also considered a junior synonym of *P.deauratus* is *P.consternans*, referred to as a variant of *P.bolovenensis* by Kottelat (2013) in an environmental assessment of a hydroelectric power project in the Bolaven Plateau, Laos and relegated to the synonymy of *P.deauratus* by Kang et al. (2016).

Morphological and molecular data also provide evidence that newly collected specimens from the Annam ecoregion represent a new species of *Poropuntius*, described below. Synonyms listed are only those addressed in this study.

### ﻿Taxonomic account

**Cypriniformes**

**Cyprinidae Rafinesque, 1815**

**Poropuntini Menon, 1999**

***Poropuntius* Smith, 1931**

#### ﻿ Poropuntius krempfi

Taxon classificationAnimaliaCypriniformesCyprinidae

(Pellegrin & Chevey, 1934)

BFB5AE11-FE32-5C55-B7BC-EB0FBF9E8BC1

Barbus (Lissochilichthys) krempfi Pellegrin & Chevey, 1934: 339. Type locality: Vietnam, Tonkin, Ngoi-Thia River at Nghia Lô, tributary of Red River upstream of Yên Bay. Holotype: MNHN 1934-0262.
Barbus (Puntius) annamensis Pellegrin & Chevey, 1936: 225, fig. 3. Type locality: Vietnam, Annam, Quảng Nam, Hàn Giang. Holotype: MNHN 1935-0337.
Lissochilus macrosquamatus Mai, 1978: 99, fig. 42. Type locality: northern Vietnam. Syntypes: DVZUT.

##### Notes.

**Figure 3.**
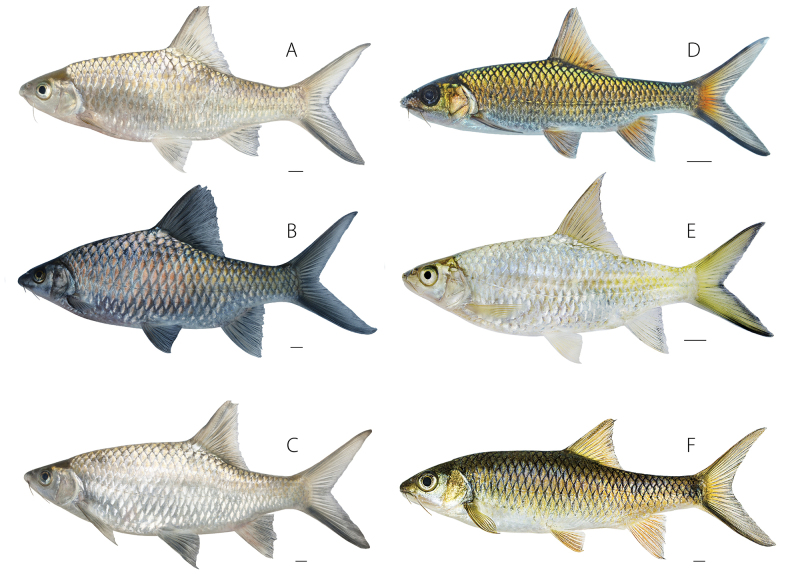
Species of *Poropuntius*. *Poropuntiuskrempfi* from **A** Song Hong ecoregion, UNS 2018-1410 **B** northern Annam, UNS 2018-2702; *P.alloiopleurus* from **C** Song Hong, UNS 2018-3010; *P.laoensis* from **D** upper Sekong’s tributary, UNS 2018-0404 **E** upper Sekong’s pool, UNS 2018-0304; *P.huangchuchieni* from **F** Lower Lancang, UNS 2018-0711. All photographed in life. Scale bars: 10 mm.

Specimens of *Poropuntiuskrempfi* (Fig. 3A, B) were recovered in a clade including topotypic material of *Barbusannamensis* and *Lissochilusmacrosquamatus*. The clade was sister to a large clade including *P.alloiopleurus*, *P.heterolepidotus*, *P.huangchuchieni*, *P.laoensis*, and *P.opisthopterus* (Fig. 2A).

Specimens of *P.krempfi* from northern Annam (MNHN 1935:0337, MNHN 1969:42) were assigned by Rainboth (1996) to *Hypsibarbusannamensis* presumably due to the presence of a few tiny tubercles on the tip of the snout, but this trait is not useful for separating all species of the two genera. Some of our 41 specimens of *P.krempfi* have tiny tubercles on the tip of the snout. Rainboth (1996) noted that this species was unusual in being the only species of *Hypsibarbus* on the coastal side of the Annam Cordillera and in having a much longer dorsal spine with 26–28 serrations, compared to 20 or fewer in other species in the Mekong River drainage.

*Hypsibarbusmacrosquamatus* from Song Hong, northern Vietnam (type: DVZUT) described by Mai (1978) and treated as *Acrossocheilusmacrosquamatus* by Nguyễn and Ngô (2001) resembles our *P.krempfi* specimens from northern Annam with a lateral-line scale count of 29–33.

##### Distribution.

*Poropuntiuskrempfi* is found in the Song Hong and northern Annam ecoregions.

#### ﻿ Poropuntius alloiopleurus

Taxon classificationAnimaliaCypriniformesCyprinidae

(Vaillant, 1893)

B3B91149-CA64-57BC-A694-7E160E010B9F

Barbus alloiopleurus Vaillant, 1893: 201. Type locality: Vietnam, Tonkin, Black River. Holotype: MNHN 1892-0261.
Barbodes (Barbodes) huangchuchieni rhomboides Wu & Lin, in Wu et al. 1977: 248, pl. 7–8. Type locality: China, Yunnan, Yuanjiang River. Syntypes (15): KIZ 645614-15, 645617-19, 645622, 645624, 6440093, 6440392, 6440461, 6440564, 6450172, 6450191, 6450207, 6450322.
Poropuntius baolacensis (Nguyen, in Nguyễn and Ngô 2001): 387, fig. 189. Type locality: Vietnam, Cao Bang Province, Nho Que, Bao Lac [22°56'60"N, 105°40'00"E], Song Hong River drainage. Holotype: VNCNTTS H.01.72.13.01.
Poropuntius brevispinus (Nguyễn & Đoàn, 1969):12, fig. 7. Type locality: Vietnam, Hoa Binh Province, Suoi Rut stream. Syntypes (3): NCNTTSI H.01.72.10.01-03.
Acrossocheilus macrophthalmus Nguyen, in Nguyễn and Ngô 2001: 390, fig. 191. Type locality: Vietnam, Hoa Binh Province, Da Bac District, Da River at Thac Bo. Holotype: NCNTTSI H.01.72.14.01.
Acrossocheilus xamensis Kottelat, 2000: 38, fig. 1. Type locality: Laos, Houaphan Province, Houay Tangoua, small stream entering Nam Xam in Ban Houay Tangoua, 20°09'24"N, 104°32'50"E. Holotype: ZRC 45297.

##### Notes.

Specimens of *Poropuntiusalloiopleurus* (Fig. 3C) were recovered in a clade including topotypic material of *Acrossocheilusmacrophthalmus*, *A.xamensis*, *Barbodeshuangchuchienirhomboides*, *Poropuntiusbaolacensis*, and *P.brevispinus* and was sister to a clade including *P.heterolepidotus*, *P.huangchuchieni*, and *P.laoensis* (Fig. 2A).

Kottelat (2000) noted that *Acrossocheilusxamensis* resembles *Poropuntius* but lacks the branched lateral-line canals diagnostic of *Poropuntius*. Our 12 specimens of *P.alloiopleurus* are missing branched lateral-line canals; branched canals are variable within *Poropuntius* and do not distinguish *Poropuntius* from *Acrossocheilus*. A paratype of *A.xamensis* exhibits the typical caudal-fin characteristic of *Poropuntius*, a dark marginal stripe along the lower lobe (Kottelat 2000, fig. 1).

According to Nguyễn (2001), *A.macrophthalmus* resembles *P.alloiopleurus* but has an eye diameter longer than the snout. Our 12 specimens of *P.alloiopleurus* have eye diameters that are shorter or longer than the snout.

Nguyễn and Ngô (2001) distinguished *P.baolacensis* from *P.brevispinus* in having 28–30 vs 18–22 serrae along the posterior margin of the last simple dorsal-fin ray. Our 12 specimens of *P.alloiopleurus* have 20–29 serrae on the last dorsal-fin ray, partially overlapping the serrae counts given to distinguish *P.baolacensis* and *P.brevispinus*.

##### Distribution.

*Poropuntiusalloiopleurus* is found in the Song Hong ecoregion.

#### ﻿ Poropuntius huangchuchieni

Taxon classificationAnimaliaCypriniformesCyprinidae

(Tchang, 1962)

154D2E92-3BCC-5D61-9949-884E2F951E9D

Barbus huangchuchieni Tchang, 1962: 96, fig. 1. Type locality: China, Yunnan, Hsi-Shuan- Pan-Na [Xishuangbanna]; Syntypes: ASIZB [now NZMC] 39256(1), 39865(1).
Poropuntius angustus Kottelat, 2000: 46, fig. 14. Type locality: Laos, Louangphabang Province, Houay Houn, ca. 3 km upstream of Ban Houay Lek, approx. 20°32'32"N, 102°40'51"E. Holotype: ZRC 45308.

##### Notes.

In the original description of *P.angustus*, Kottelat (2000) made no specific mention of *P.huangchuchieni*. Topotypic material (UNS 2018-07-11, Fig. 3F) from the Nam Nứa, Lower Lancang ecoregion, in the upper reach of Nam Ou, the type locality of *P.angustus*, was genetically identical to that of *P.huangchuchieni* from the Lancang Jiang in Yunnan.

##### Distribution.

*Poropuntiushuangchuchieni* is found in the Lower Lancang ecoregion.

#### ﻿ Poropuntius laoensis

Taxon classificationAnimaliaCypriniformesCyprinidae

(Günther, 1868)

E3627E3F-762E-5AF6-A63A-51F478AF8192

Barbus laoensis Günther, 1868: 115. Type locality: Laos Moutains, Cochinchina. Holotype: BMNH 1862.7.28.15.
Poropuntius bantamensis (Rendahl, 1920): 1, fig. 1. Type locality: Thailand, Ban Tam, east of Doi Chieng Dao, pond fed by a subterranean stream. Holotype: NRM 10969.
Poropuntius aluoiensis (Nguyen, 1997): 1, fig. 1. Type locality: Vietnam, Thua Thien Hue Province, A Luoi District, A Sap stream at Nham, Sekong basin, 16°15'32"N, 107°13'31"E. Holotype: HNUE V1.1.21.

##### Notes.

Our seven specimens of *P.laoensis* specimens (Fig. 3D, E) were collected in the upper Xekong drainage of Central Vietnam, the type locality, i.e., in the Lao Mountains of Cochin-China, now Central Vietnam. Topotypic material of *P.bantamensis* and *P.aluoiensis* resembled *P.laoensis* in having the proximal half of the caudal fin orange and the distal half bright yellow, and bold black submarginal stripes on the caudal fin.

##### Distribution.

*Poropuntiuslaoensis* occurs in the Lower Salween, Chao Phraya, Lower Lancang, northern Annam, and Kratie-Stung Treng ecoregions.

#### ﻿ Poropuntius kontumensis

Taxon classificationAnimaliaCypriniformesCyprinidae

(Chevey, 1934)

BB6CD696-6790-5ED3-9AB0-9CAF05344542

Cyclocheilichthys kontumensis Chevey, 1934: 32, fig. 1. Type locality: Vietnam, Annam, Pleiku Province, Kontum Lake [Mekong River drainage]. Types unknown.
Acrossocheilus yalyensis Nguyễn, in Nguyễn and Ngô 2001: 393, fig. 193. Type locality: Vietnam, Kon Tum Province, Sông Sesan [river]. Holotype: NCNTTSI H.01.72.15.01.

##### Notes.

**Figure 4.**
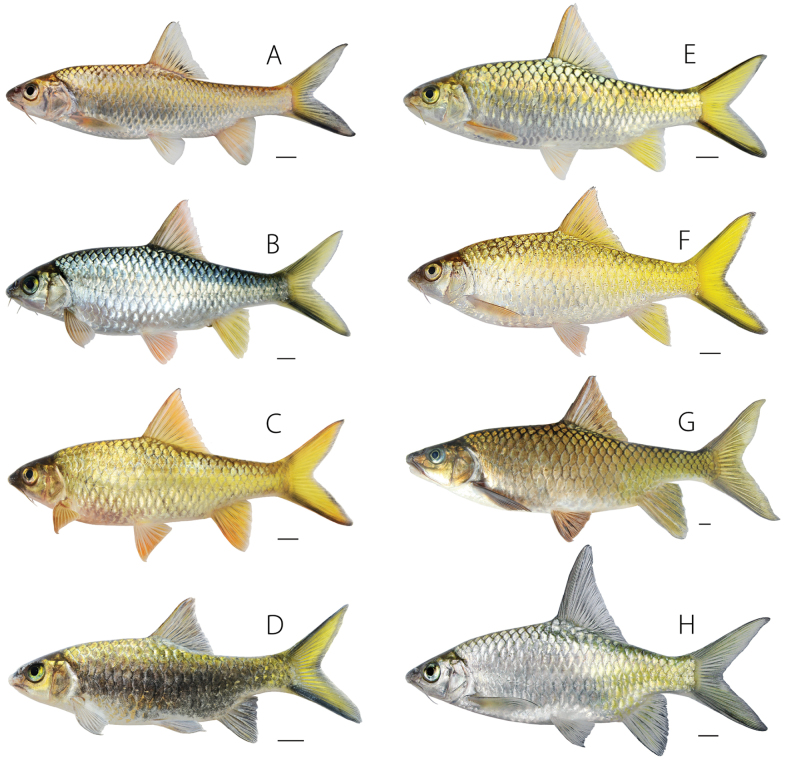
*Poropuntiuskontumensis* and *P.deauratus*. *Poropuntiuskontumensis* from **A** Kratie–Stung Treng ecoregion (the Sesan drainage), UNS 2018-2201; *P.deauratus* from **B** northern Annam, UNS 2017-0310 **C** northern Annam, UNS 2018-2502 **D** southern Annam, UNS 2018-0801 **E** Mekong Delta (the Đồng Nai drainage) UNS 2017-1612 **F** Khorat Plateau (Sepon drainage), UNS 2018-0904 **G** upper Srepok drainage, UNS 2015-2301 **H** lower Srepok drainage’s pool, UNS 2015-3006. All photographed in life. Scales bars: 10 mm.

The five specimens of *Poropuntiuskontumensis* (Fig. 4A) and *Acrossocheilusyalyensis* were collected in the Kratie – Stung Treng ecoregion from their type localities and were genetically identical.

##### Distribution.

*Poropuntiuskontumensis* occurs in the Kratie-Stung Treng ecoregion.

#### ﻿ Poropuntius deauratus

Taxon classificationAnimaliaCypriniformesCyprinidae

(Valenciennes, in Cuvier & Valenciennes, 1842)

9C7E66A1-3362-5C04-85D0-B00FCE068526

Barbus deauratus Valenciennes, in Cuvier and Valenciennes 1842: 188. Type locality: Vietnam, Cochinchina [South of Hué; Kottelat 2000: 45]. Holotype: MNHN 2727.
Poropuntius normani Smith, 1931: 15. Type locality: Thailand, Chantaburi Province, Nam Tok Pliu, Kao Sabap, near Chantaburi. Holotype: USNM 90297.
Poropuntius smedleyi de Beaufort, 1933: 44. Type locality: Malaysia, Johor. Syntypes: ZRC [1, missing], ZMA 101.007 [Nijssen et al. 1993: 214).
Poropuntius bolovenensis Roberts, 1998: 124, fig. 5. Type locality: Laos, Champasak Province, Bolavens Plateau, Sekong basin, Xe Nam Noi, 300 m downstream from primary dam site of Xe Nam Noi–Xe Pian hydropower scheme. Lectotype: CAS 94251 [designated by Kottelat 2000: 46].
Poropuntius lobocheiloides Kottelat, 2000: 48. Type locality: Laos, Champasak Province, Bolavens Plateau, Xe Nam Noi 300 m downstream from primary dam site for Xe Nam Noi–Xe Pian hydropower scheme, Bolavens Plateau, Sekong watershed. Holotype: CAS 94257.
Poropuntius solitus Kottelat, 2000: 48, fig. 15. Type locality: Laos, Champasak Province, Bolavens Plateau, Huay Makchan-Gnai (Xe Nam Noy basin), south of Ban Taot at turnoff to Xe Nam Noy Project, on road from Pakse to Attapu, 15°04'14"N, 106°32'33"E. Holotype: ZRC 45310.
Poropuntius consternans Kottelat, 2000: 48. Type locality: Laos, Champasak Province, Bolavens Plateau, Sekong drainage, Xe Nam Noi, 300 m downstream from primary dam site of Xe Nam Noi–Xe Pian hydropower scheme, Bolavens Plateau, Sekong watershed. Holotype: CAS 94255.

##### Notes.

The *P.deauratus* clade contained 56 specimens of six nominal species, *P.normani*, *P.deauratus*, *P.bolovenensis*, *P.lobocheiloides*, *P.smedleyi*, and *P.solitus*, collected from a wide area including the Eastern Gulf of Thailand drainages, Malay Peninsula, Mekong Delta, Kratie – Stung Treng, Khorat Plateau, and Annam ecoregions, including type localities.

Molecular data from specimens originally identified as *P.bolovenensis*, *P.lobocheiloides*, and *P.solitus* (GenBank: Song et al. 2017) are from specimens identified morphologically by Kang et al. (2016), who also discussed variation in these forms and in *P.consternans*. Kang et al. (2016) considered all to be *P.bolovenensis*. Our results agree with the conspecificity demonstrated by Kang et al. (2016) but place *P.bolovenensis* in the synonymy of *P.deauratus*. Roberts (1998) had correctly treated *P.consternans* and *P.lobocheiloides*, later named by Kottelat (2000), as ecomorphs of *P.bolovenensis*. Our five specimens of *P.normani* were collected in the same small area as the type locality.

*Poropuntiusdeauratus* occurs over a large geographic area and shows considerable variation in shape and color (Fig. 4B–H) as well as in the structural traits related to feeding morphology.

##### Distribution.

*Poropuntiusdeauratus* is found in the Western Malaysia, Malay Peninsula Eastern Slope, Eastern Gulf of Thailand, Mekong Delta, Kratie-Stung Treng, Khorat, northern Annam, and southern Annam ecoregions.

#### ﻿ Poropuntius anlaoensis

Taxon classificationAnimaliaCypriniformesCyprinidae

Hoàng, Phạm & Trần sp. nov.

3A9B2F5F-5662-5965-ACE8-6975551502D1

https://zoobank.org/17935DFC-446B-4F5C-AF44-4719D31BAD3C

##### Material examined.

**Figure 5.**
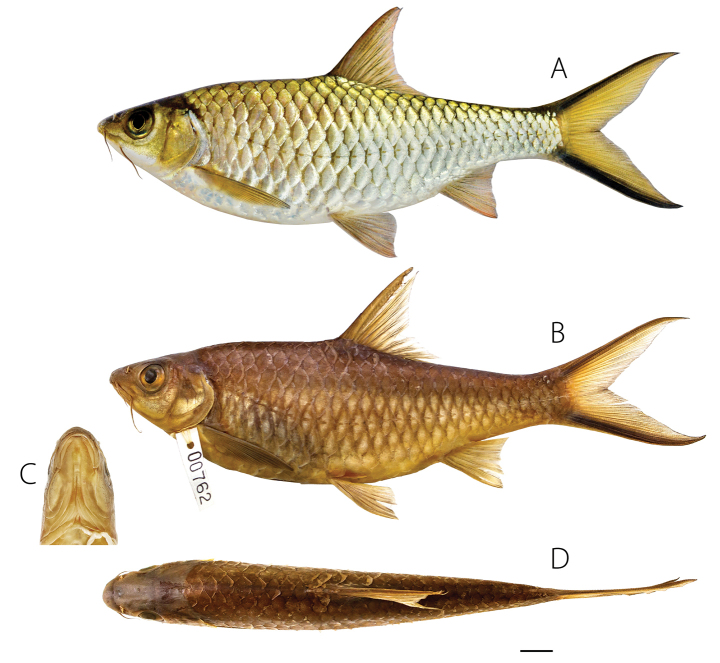
*Poropuntiusanlaoensis***A** adult holotype (UNS00762, 139.46 mm SL) in life **B** in preservative **C** ventral view of head **D** dorsal view. Scale bars: 10 mm.

***Holotype***: UNS00762, 139.46 mm SL, female; An Lão drainage, Bình Định Province, Vietnam (14°40'30.6"N, 108°54'13.4"E, 547 m), 19 January 2013, Hoàng Đức Huy, Phạm Mạnh Hùng and Trần Trọng Ngân (Fig. 5). ***Paratypes***: An Lão drainage, Bình Định Province, Vietnam (14°40'30.6"N, 108°54'13.4"E): UNS 2013-19-01, 5 specimens, 104–139 mm SL, 19 January 2013; Re River, Sơn Hà, Quảng Ngãi Province, Vietnam (15°01'03.9"N, 108°31'55.0"E): UF 249109, 5 specimens, 99–150 mm SL, 24 February 2018.

##### Diagnosis.

*Poropuntiusanlaoensis* is the only species of the genus found on the coastal side of the Annamite Cordillera and nowhere else. It differs from all other species of *Poropuntius* genetically (Fig. 2A), and by having distal margin of dorsal fin distinctly concave (vs straight to slightly concave). It most closely resembles *P.deauratus* but has 29–31 (vs 25–28) lateral-line scales, snout distinctly pointed (vs slightly pointed), and caudal fin light yellow with bold black submarginal stripes (vs bright lemon yellow to dusky with bold to faint black submarginal stripes). *Poropuntiusanlaoensis* differs from *P.genyognathus*, *P.hampaloides*, and *P.melanogrammus* in having barbels (vs no barbels); from *P.heterolepidotus* and *P.hathe* in having scales on posterior half of body not markedly smaller (vs markedly smaller) than those on anterior half; from *P.alloiopleurus*, *P.burtoni*, *P.carinatus*, *P.huangchuchieni*, *P.kontumensis*, *P.krempfi*, *P.laoensis*, *P.opisthoptera*, and *P.schanicus* in having 29–31 (vs > 32) lateral-line scales and bold black submarginal stripes (vs no bold black stripes) on caudal fin.

##### Description.

General appearance in Fig. 5; meristic and morphometric data of 11 specimens in Table 3. Head conical, longer than deep, depth 1.2–1.4 × in HL. Snout pointed. Tubercles tiny and few on tip of the snout, many irregular transverse rows of small tubercles reaching front of eyes in male. Mouth subterminal and oblique, extending posteriorly in length slightly longer than eye diameter and broadly horseshoe-shaped (Fig. 5C). Rostral barbel shorter than maxillary barbel, both longer than eye diameter.

**Table 3.** Morphometric and meristic characters of *Poropuntiusanlaoensis* sp. nov., *n* = 11 including holotype.

	*Poropuntiusanlaoensis*		
	Holotype UNS00762	Range	Mean ± SD / mode
**SL (mm)**	139.46	99.2–150.9	118.6 ± 16.4
**Morphometrics**			
% **SL**			
Total length	127.6	124.8–129.3	127.1 ± 1.4
Fork length	108.1	107.2–109.7	108.3 ± 0.6
Body depth	31.4	29.9–35.7	31.6 ± 1.5
Body width	13.5	13.5–16.3	14.8 ± 0.7
Head length	24.4	22.4–25.7	24.4 ± 1.2
Caudal peduncle length	22.5	18–23.9	21.1 ± 1.6
Caudal peduncle depth	10.9	10.9–13.4	11.9 ± 0.7
Dorsal-fin base length	15.6	15.6–18.8	16.2 ± 0.9
Dorsal-fin length	25.8	21.4–29.7	25.3 ± 2.1
Anal-fin base length	8.9	8.4–10.5	9.3 ± 0.6
Anal-fin length	16.2	14.8–21.3	16.5 ± 1.8
Pectoral-fin length	22.1	20.2–22.6	21.4 ± 0.8
Pelvic-fin length	18.7	16.8–20.9	18.8 ± 1.1
Predorsal length	48.8	44.5–49.4	48.1 ± 1.2
Prepectoral length	21.3	19.8–23.7	21.6 ± 1
Preanal length	72.2	69.7–75.2	72.5 ± 1.6
Prepelvic length	48.0	46.5–50.8	48.6 ± 1.4
Pelvic-fin base to anal-fin base	22.0	18.8–22.8	21.1 ± 1.1
Pectoral-fin base to pelvic-fin base	25.5	21.3–26.2	23.4 ± 1.6
% **HL**			
Head depth at nape	84.3	44.5–84.3	76.6 ± 10.7
Head width	56.7	54.8–63.7	58.3 ± 2.5
Snout length	30.8	26.3–35.8	29.5 ± 2.6
Interorbital width	35	32.9–41.9	37.9 ± 2.7
Eye diameter	23.5	23.5–27.3	25.9 ± 1.1
Mouth width	30.8	25.4–33.2	29.6 ± 2.5
Rostral barbel length	38.2	23.1–38.2	30.4 ± 4
Maxillary barbel length	31.4	23.3–39	33.4 ± 4.3
**Counts**			
Dorsal-fin spines and rays	iv,8.5	iv,8.5	
Anal-fin spines and rays	iii,5.5	iii,5.5	
Pectoral-fin spines and rays	i,15	i,11–16	mode = i,15
Pelvic-fin spines and rays	i,8	i,8	
Lateral-line scales	29	29–31	mode = 30
Transverse scales rows above lateral-line	5.5	5.5	
Transverse scales rows below lateral-line	3.5	3.5	
Circumferential scale rows	20	20–22	mode = 22
Circumpeduncular scale rows	14	14	
Predorsal scales	11	10–12	mode = 10
Scales from end of anal-fin base to caudal-fin origin	8	7–9	mode = 7
Serrae on last simple dorsal-fin ray	24	16–27	mode = 24

Body moderately deep and compressed, depth approximately 2.8–3.3 × in SL. Dorsal body profile convex, slightly convexity from nape with narrow dorsum almost straight in front of dorsal origin to dorsal fin. Base dorsal fin decreasing in height nearly straight dorsal margin of the body, extending from dorsal-fin origin to narrowest part of the caudal peduncle. Ventral profile rounded, rising through anal-fin insertion to caudal-fin base. Caudal peduncle slender, moderately shallow and long, 1.6–2.1 × longer than deep. Anus immediately in front of anal fin. Lateral line complete, 29–31 scales; 10–12 predorsal scales; 5/1/3 scales in transverse row anterior to pelvic-fin origin. Lateral-line tubes extending at least halfway across each scale, with accessory pore on ventral branch on nearly every lateral-line scale. Dorsal iv-8.5, pectoral i-15, pelvic i-8, and anal iii-5.5.

Dorsal fin high and sharply pointed at apex, last unbranched ray longest, followed by first branched ray which is considerably shorter. Last unbranched ray ossified with 18–20 serrae. Posterior extensions of serrae forms straight line, with base line curved posteriorly. More distal denticles curved along their lengths. Distal margin of fin strongly concave, with posteriormost ray equalling length of third branched ray. Dorsal-fin origin approximately opposite pelvic-fin origin. Dorsal-fin base longer than anal-fin base.

Pectoral fin long, extending to third scale row before pelvic-fin origin.

Pelvic fin not extending to base of last unbranched anal ray. Distal margin concave near tip with falcate apex. Axillary scale present at base of pelvic fin.

Anal fin moderate, distal margin straight when fin is erect.

Caudal fin deeply forked with outer rays nearly 4 × length of middle rays. Upper and lower lobes nearly equal in length with straight distal margin on each lobe.

##### Colour in life.

Head dark greenish golden on top, greenish golden around orbit and on opercula, white on lower jaw. Body primarily silvery but greenish golden on dorsally to lateral line, scale bases with melanophores. All fins except caudal hyaline with pinkish orange tinge on branched rays; fins more darkly pigmented in adults. Caudal fin yellow with bold black submarginal stripes (Fig. 5A).

##### Colour in preservative.

Body including head dark brown on back. Opercula dark at base. Lower half of body light brown. Scale margins lined with brown, forming network. All fins brown to dark brown (Fig. 5B).

##### Etymology.

Specific epithet is in reference to the type locality, the An Lão drainage.

##### Suggested common name.

Cá hồng nhau An Lão (Vietnamese), Anlao brook barb (English).

##### Distribution.

*Poropuntiusanlaoensis* is restricted to the southern Annam ecoregion and is possibly endemic to the coastal side of the Annamite Cordillera.

### ﻿Key to species of *Poropuntius*

**Table:** 

1	No barbels; black on distal half of dorsal fin	**2**
–	Maxillary barbels present; black, if present, confined to margin of fin	**3**
2	Bold black midlateral stripe on body	***P.melanogrammus***
–	No black midlateral stripe on body	***P.hampaloides***
3	No rostral barbels; maxillary barbels very small (nubs)	***P.genyognathus***
–	Rostral and maxillary barbels present	**4**
4	Scales on posterior half of body markedly smaller than those on anterior	**5**
–	Scales on posterior half of body not markedly smaller than those on anterior	**6**
5	Lateral-line scales 39–40; no black submarginal stripes on caudal fin	***P.heterolepidotus***
–	Lateral-line scales 33–35; bold black submarginal stripes on caudal fin	***P.hathe***
6	Lateral-line scales 35–46; branched lateral-line canals almost absent	***P.alloiopleurus***
–	Lateral-line scales ≤ 40; branched lateral-line canals present	**7**
7	No black submarginal stripes on caudal fin	**8**
–	Black submarginal stripes on caudal fin	**10**
8	Lateral-line scales 29–31; barbels short and thin, rostral barbel not extending to anterior margin of eye	***P.schanicus***
–	Lateral-line scales > 35; barbels well developed, rostral barbel extending to anterior margin of eye	**9**
9	Predorsal scales 12–14	***P.burtoni***
–	Predorsal scales 15–16	***P.opisthoptera***
10	Lateral-line scales < 32 or > 32 with caudal fin bright yellow to dusky greenish yellow	**11**
–	Lateral-line scales > 32 without caudal fin colour as above	**12**
11	Head longer than deep; distal margin of dorsal fin distinctly concave	***P.anlaoensis***
–	Head shorter than deep; distal margin of dorsal fin straight	***P.deauratus***
12	Bold black marginal stripes on caudal fin; green-gold to silver scales; orange to orangish yellow caudal fin	***P.laoensis***
–	Dusky grey or brown marginal stripes on caudal fin	**13**
13	Dorsal fin height ≥ body depth at dorsal-fin origin	***P.carinatus***
–	Dorsal fin height < body depth at dorsal-fin origin	**14**
14	Caudal fin dusky, grey to black	***P.krempfi***
–	Caudal fin orange to yellow-orange at base or very pale yellow	**15**
15	Silver scales on body; Lower Lancang ecoregion	***P.huangchuchieni***
–	Orange-bronze scales on body; Kratie-Stung Treng ecoregion	***P.kontumensis***

## ﻿Discussion

The objective of this study was to test, using molecular data, the species diversity of *Poropuntius*, especially that of Vietnam in which 15 species names have been recognised recently as valid. As noted above, most species of *Poropuntius* have been described using only morphological data, and recent investigations using molecular data suggested that some morphological hypotheses were incorrect. Our data indicate that 17 names assigned to species are synonyms.

Several factors led to the inflation of species names for *Poropuntius*. These include limited efforts overall in collecting and classifying cyprinid fishes, especially in northern Vietnam, limited access to type specimens in European museums, limited availability of publications (often in foreign languages – although now this is being alleviated by online access), inadequate descriptions containing limited information, and failure to appreciate morphological variation related to trophic diversification within species. Finally, a substantial portion of the cyprinid diversity of northern Vietnam is shared with that of southern China, but researchers in the two countries have not had access to specimens from both countries or to original descriptions in other languages. Consequently, studies were limited to in-country fauna, and species in each country were treated as endemic to that country.

Ecomorphological variation in *Poropuntius* was described and figured by Roberts (1998: figs 6, 8), and Kang et al. (2016) interpreted *P.bolovenensis*, *P.lobocheiloides*, and *P.solitus* as ecomorphs of *P.bolovenensis*. The often-extreme intraspecific phenotypic variation in the shape of the mouth and lips, and in the development of the horny sheath on the lower jaw, has led to taxonomic confusion in *Poropuntius*. Ecophenotypic variation is expressed especially clearly in species occupying pools isolated by barriers such as waterfalls in streams on plateaus at high elevations (e.g., on the Bolaven, Kontum, and Langbiang Plateaus) and in drainages leading to the Gulf of Tonkin (in the Song Hong and Annam ecoregions). Species not exhibiting such extreme polymorphism may nevertheless carry information in their genomes necessary to generate an array of potentially taxonomically confusing, continuous variation as well as more disjunct phenotypes. For example, the position of the dorsal fin, often used in taxonomic diagnoses, varies from more anteriorly to more posteriorly positioned in *Poropuntius*, as seen in *P.alloiopleurus*, *P.huangchuchieni*, and *P.laoensis* (Wu et al. 2013; this study). Specimens of *Poropuntius* from flowing water habitats are characterised by a more elongated body and a relatively long caudal peduncle, while those from pools have a deeper body and larger unpaired fins (Figs 3A, B, D, E, 4G, H). Similar patterns have been described in other cyprinids, e.g., *Rasborapaviana* and *Lobocheilosrhabdoura*, with populations inhabiting fast-flowing streams having more slender bodies than those inhabiting slower-flowing habitats (Stolbunov et al. 2011; Ciccotto and Page 2016).

During a decade of field work on Southeast Asian freshwater fishes, the first author and colleagues have collected samples of *P.deauratus*, *P.krempfi*, *P.alloiopleurus*, and *P.laoensis* revealing marked intraspecific phenotypic variation. These samples include those from river basins in which few or no fish collections have been made or reported upon previously, and which fill in gaps in the distributions of these species. Pronounced variation among adult individuals in these species has been observed in body depth, dorsal-fin spine length, ossification and serration of the last simple dorsal-fin ray, body colour, and caudal fin colour. For example, in the wide-ranging *P.deauratus*, the typical colour in life is a silvery to light green gold body with bright yellow on the posterior half of the body and a bright lemon-yellow caudal fin with bold black submarginal stripes. However, colour in this species may be much more subtle with the body uniformly dusky to dark and the fins dusky (Fig. 4B, H).

## ﻿Conclusions

This study has revealed that scientific names have been applied erroneously to populations of *P.krempfi*, *P.alloiopleurus*, *P.huangchuchieni*, *P.laoensis*, *P.kontumensis*, and *P.deauratus*. Errors in the taxonomy of *Poropuntius* have resulted primarily from inadequate sampling and reliance on characters that vary intraspecifically. Additional samples from unsampled or poorly sampled populations allowed the use of molecular data to test previous species hypotheses. More sampling and use of molecular as well as morphological data on species of *Poropuntius* that could not be included in this study are likely to require additional taxonomic changes.

## Supplementary Material

XML Treatment for Poropuntius krempfi

XML Treatment for Poropuntius alloiopleurus

XML Treatment for Poropuntius huangchuchieni

XML Treatment for Poropuntius laoensis

XML Treatment for Poropuntius kontumensis

XML Treatment for Poropuntius deauratus

XML Treatment for Poropuntius anlaoensis

## ﻿Additional information

### Author ORCIDs

Huy Duc Hoang https://orcid.org/0000-0001-6528-193X

Hung Manh Pham https://orcid.org/0000-0003-3186-818X

Ngan Trong Tran https://orcid.org/0000-0001-7254-2835

Jean-Dominique Durand https://orcid.org/0000-0002-0261-0377

Wu Ling https://orcid.org/0009-0001-5700-1616

John Pfeiffer https://orcid.org/0000-0001-5368-0589

Xiao-Yong Chen https://orcid.org/0000-0002-0924-5560

Lawrence M. Page https://orcid.org/0000-0002-8823-0729

### Data availability

Voucher specimens are available as described in the text and sequence data are posted on GenBank.

## Supplementary materials

Supplementary material 1 Samples included in the molecular analysis, with GenBank accession numbers Data type xlsx Explanation note **table S1.** Samples included in the molecular analysis, with GenBank accession numbers for Cytochrome oxidase c subunit 1 (COI) and Cytochrome *b* (Cyt*b*) sequence data. https://binary.pensoft.net/file/1063265This dataset is made available under the Open Database License (http://opendatacommons.org/licenses/odbl/1.0/). The Open Database License (ODbL) is a license agreement intended to allow users to freely share, modify, and use this Dataset while maintaining this same freedom for others, provided that the original source and author(s) are credited.Huy Duc Hoang, Hung Manh Pham, Ngan Trong Tran, Jean-Dominique Durand, Wu Ling, John Pfeiffer, Xiao-Yong Chen, Lawrence M. Page
